# Artemisinin-Based Combination Therapy Depressed Mitosis and Induced Chromosome Aberration in Onion Root Cells

**DOI:** 10.1155/2018/4671326

**Published:** 2018-08-23

**Authors:** J. I. Raji, C. K. Onwuamah, P. G. C. Odeigah

**Affiliations:** ^1^Department of Cell Biology and Genetics, University of Lagos, Nigeria; ^2^Centre for Human Virology and Genomics, Nigerian Institute of Medical Research, Lagos, Nigeria

## Abstract

Artemisinin-based combination therapy is used to treat uncomplicated malaria disease in most endemic countries. Although most antimalarial drugs are effective in killing the parasite, there is a concern of induced toxicity to the cell. Here, the cytogenotoxicity of dihydroartemisinin-piperaquine phosphate (DHAP), a coformulation for artemisinin-based combination therapy, was evaluated using* Allium cepa *model. The toxicity on the mitotic index varies with the duration of exposure and dose tested. Chromosome aberrations observed include chromosome fragments, chromosome bridges, binucleated cells, and micronucleated cells. This study showed that DHAP can depress mitosis and induce chromosome abnormalities. Their accumulation in cells may be inhibitory to cell division and growth. This calls for caution in the administration of artemisinin combination therapy for the treatment of malaria ailment. Wide spacing of dosage is therefore suggested in order to avoid the risk of genetic damage.

## 1. Introduction

Currently, artemisinin-based combination therapy (ACT) is widely used as the first-line treatment of uncomplicated malaria in most African countries [1, 2]. It has proven to be effective in killing the immature sexual stages of malaria parasites [3]. The provision of ACT for treatment of uncomplicated malaria has been associated with reduced* Plasmodium* parasite burden in different populations. In addition, low intensity of disease transmission has been reported in diverse settings that used ACT [4, 5]. The efficacy of ACT has been reported to extend to phylogenetically non-related parasitic infections such as trypanosomiasis [6]. They have also shown potent antiviral [7], antifungal [8], and broad anticancer properties in cell lines and animal models [9]. Notwithstanding its potency as a gametocytocidal agent, most of the drugs targeted at killing* Plasmodium *parasite have been shown to induce toxicity to the host cell [10]. In addition, some plant extracts used in the treatment of uncomplicated malaria have been shown to be toxic to the adult mosquito population [11]. Recently, ACT has been implicated in neuroauditory toxicity [12]. Similarly, dihydroartemisinin (DHA) has been reported to arrest growth in the G1 phase of hepatoma cell lines [13] and disrupted the cell cycle at G2/M in pancreatic cancer cell lines [14]. No cytogenotoxicity study has been reported for piperaquine. However, it has been shown to be non-teratogenic in pregnant rats [15]. Plant root tips have been widely used to evaluate and monitor cytotoxicity of chemical substances [16, 17]. Therefore, this study tested the cytogenotoxic potential of dihydroartemisinin-piperaquine phosphate (DHAP) using the meristematic cells of* Allium cepa*.

## 2. Materials and Methods

### 2.1. Drug Preparation

Antimalarial drug containing the combination of dihydroartemisinin (80mg) and piperaquine phosphate (640mg) per 80ml was purchased from a local pharmaceutical store in Nigeria. Small volume of deionized water was added to make up 80ml suspension as directed by the manufacturer. Serial dilution of 80mg/80ml stock of dihydroartemisinin was prepared with deionized water which served as the diluent to obtain varying doses needed for the treatment.

### 2.2. Preparation of Test Organism

Purple variety of* Allium cepa *bulbs used for this study was purchased from Mile 12 International Market, Lagos, Nigeria. Physical screening was done to sort out wholesome ones and measurements such as weight (50± 3cm) and diameter (5.5± 2cm) were recorded. The bulbs were reeled off their old brownish scales and the old roots were cut off, while the primordial roots were left intact.

A total of twenty onion bulbs were selected for pretreatment. The basal part of the onion bulb was placed in contact with clean tap water contained in a 35ml capacity bottle (diameter 3.5cm) to achieve sprouting. The set-up was maintained at a temperature of 25± 1°C and relative humidity of 54± 1% for 24 hours with alternation of light (9.00 a.m. to 6.00 p.m.) and dark cycle (6.00 p.m. to 9.00 a.m.).

### 2.3. The 96-Hour Root Growth Inhibition Test

The method used in this test was adopted from [18, 19] with slight modifications. The 96-hour root growth inhibition was performed first to determine the EC_50_ and thus obtain a good range for the test organism. The half maximal effective concentration (EC_50_) refers to the concentration which can induce a response halfway between the baseline and the maximum over a specified period of time. With respect to this assay, the EC_50_ would represent the concentration of the drug that would allow 50% of the root growth achieved by the untreated controls. This was determined as 96-hour semistatic exposure test using 6 concentrations of each drug. A total of thirty test organisms were used for range finding. The root lengths of the bulbs were measured with range of (24± 0.2mm) and were selected for the* Allium cepa* test. Five groups including the negative control were set up with six replicates each. The tap water in each bottle was replaced with 35ml of graded concentrations (1.5, 25, 75, and 150*μ*g/ml) prepared from the stock solution by serial dilution. At the therapeutic dose, the dihydroartemisinin human plasma levels are equivalent to 1.5*μ*g/ml. This helped select the starting dose for determining the EC_50_.

The bulbs were grown away from direct sunlight in an inner laboratory using glass bottles each holding about 35 ml of solvent. The solution was homogenized twice daily using a glass rod stirrer at the hour of nine and six to ensure aeration. At the end of the 96-hour exposure period, one* Allium cepa* bulb (out of six) with the poorest root growth was discarded; this was done to reduce outlier effect and increase the chances that an effect seen is due to the drug administered. The longest of the root bundle of the remaining five* Allium cepa* bulbs was measured using a pair of dividers and ruler. The individual root length for each replicate was recorded and averaged. The set-up was maintained at temperature of 25± 2°C and relative humidity of 54± 1% for 96 hours with alternation of light (9.00 a.m. to 6.00 p.m.) and dark cycle (6.00 p.m. to 9.00 a.m.). The effect of dihydroartemisinin on root growth was investigated by comparing the concentrations and their corresponding root length and then expressed as a percentage of the root length of the negative control.

### 2.4. Acute Toxicity Test

Test doses (1, 0.5 and 0.25 *μ*g/ml) were obtained from the EC_50_ curve, calculated from the 96-hour root growth inhibition test. After the completion of the pretreatment process, the bulbs were grown in the various doses of the dihydroartemisinin for 3 hours only. The negative control (NC) group was grown in tap water for the same duration. The set-up was maintained at temperature of 25± 1°C and relative humidity of 54± 1% with alternation of light (9.00 a.m. to 6.00 p.m.) and dark cycle (6.00 p.m. to 9.00 a.m.). After the period of exposure elapsed, all the bulbs from the test materials were removed, gently rinsed, and grown again in tap water. The root tips samples were obtained from all replicates per dose at 12h, 24h, and 48h postexposure. The roots were preserved in a freshly prepared fixative (Methanol-Glacial Acetic Acid; 3:1 v/v) and kept cool at 4-8°C pending slides preparation Ma* et al*. [20].

### 2.5. Chronic Toxicity Test

Test doses (1, 0.5 and 0.25 *μ*g/ml) were obtained from the EC_50_ curve, calculated from the 96-hour root growth inhibition test. After the completion of the pretreatment process, the bulbs were grown in the various doses of the dihydroartemisinin for 48 hours only. The negative control (NC) group was grown in tap water for the same duration. The set-up was maintained at temperature of 25± 1°C and relative humidity of 54± 1% with alternation of light (9.00 a.m. to 6.00 p.m.) and dark cycle (6.00 p.m. to 9.00 a.m.). The root tips samples were obtained from all replicates per dose at 48h post-exposure. The roots were preserved in a freshly prepared fixative and kept cool at 4-8°C pending slides preparation.

### 2.6. Test for Micronucleus (MCN)

The procedure by Ma et al. (1995) was strictly followed for this study. Test doses (1, 0.5, and 0.25 *μ*g/ml) were obtained from the EC_50_ curve, calculated from the 96-hour root growth inhibition test. After the completion of the pre-treatment process, the bulbs were grown in the various doses of DHA for 6hrs only at room temperature of 25± 1°C and relative humidity of 54± 1% with alternation of light (9.00 a.m. to 6.00 p.m.) and dark cycle (6.00 p.m. to 9.00 a.m.). After the exposure period has elapsed, the bulbs were transferred into clean tap water and root tip samples from all replicates were obtained at 24h and 44h postexposure. The roots were preserved in a freshly prepared fixative and kept cool at 4-8°C pending slides preparation.

### 2.7. *Allium cepa *Recovery Studies

The onion roots were transiently grown in the test drug solutions for 3hrs and 6hrs and later removed. Thereafter, it was transferred into a bottle containing deionized water to recover [19]. Data was scored 12hr, 24hr, and 48hr postexposure of the root length. The mean mitotic index was scored and repeated for all the replicates. Root tips were harvested and fixed for microscopic evaluation. Changes were observed in the root tips length and root growth recovery was scored.

### 2.8. Fixation and Staining of Onion Root Tips

Roots tips were fixed in freshly prepared fixative (methanol versus glacial acetic acid 3:1). In order to prepare the root tips smears, they were transferred from the refrigerator to room temperature. Hydrolysis of the root tips was carried out in 1 N HCl for 5-6 minutes. After hydrolysis, excess HCL was drained. The terminal developing root tips of 2 mm length were cut with a sharp razor and squashed on clean slides with the pointed end of a forceps. Squashing techniques described by [21] were used in preparing slides.

The slide was then stained in a lactic aceto-orcein stain and was allowed to stay for 20 min at room temperature. When staining was completed, a coverslip was carefully placed on the slide from an angle of 45° in order to ensure even spreading. The slide was held through several folds of blotting paper with pressure applied on the coverslip to expel excessive fluids and air bubbles. The edges of the slides were sealed up with nail varnish to reduce fluid evaporation.

### 2.9. Microscopic Observation

Optical microscope (Olympus-CH20) was used to view the slides. The slides were viewed for cell count at X400 magnification, while photomicrograph was taking at X1000 magnification under oil immersion. Slides per dose (replicates=5) were prepared and 500-850 cells were counted using a manual counter and examined per slide under the microscope. Interphase cells were also scored for the presence of micronucleus and binucleated cells. The observed cells were scored for the different cell division stages (interphase, prophase, metaphase, anaphase, and telophase). Total dividing cells and number of cells showing chromosome aberrations under the microscope fields were recorded. Mitotic index and percentage chromosome aberration were calculated for each treatment as previously described by Adegbite et al. [22].

## 3. Results

### 3.1. DHAP Inhibits the Root Growth of* A. cepa*

**Figure 1:**
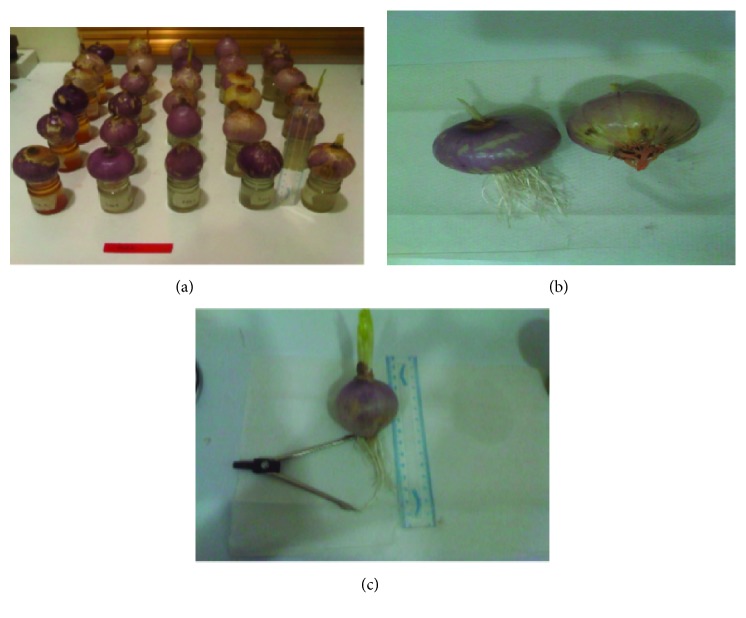
(a) Assay set-up showing onion root tips treated with different concentrations of DHAP. (b) Picture showing onion bulbs with normal (left) and inhibited (right) root growth after being treated with DHAP. (c) Picture showing how onion root tips were measured with the aid of a ruler and divider.

The results of the* Allium cepa* root growth when treated with different concentrations of dihydroartemisinin-piperaquine (DHAP) are presented in Table 1 and Figure 1. The result obtained 96h postexposure implicated DHAP as a root growth inhibitor. However, its toxicity has been observed to be dose-independent. There was no significant difference (P>0.05) between the 1.5*μ*g/ml and 75*μ*g/ml as well as the 25*μ*g/ml and 150*μ*g/ml when analysed using Turkey's test. A significant difference (P<0.05) was observed between the control and the treatments (Figure 1). The estimated EC_50_ of dihydroartemisinin (DHA) extrapolated from Table 1 is 1*μ*g/ml.

### 3.2. Toxicity of DHAP on Root Cell Mitosis

The mitotic index (MI) observed after acute exposure was time and dose-dependent.  Table 2 showed the mean values of mitotic activities observed after exposure of* A. cepa* root tips to varying doses of DHAP. As shown on Table 2, the mitotic index decreased significantly at 12hr postexposure (P=0.0194) and 24hr postexposure (P=0.021) with increase in concentration of DHAP at different duration. The mean MI in the highest treatment dose (1*μ*g/ml) increased from 2.22% to 3.02% after acute exposure. However, as shown in Table 3, a significant recovery was recorded 48hr after acute exposure as the MI of the highest concentration was not significantly different from the control (P=0.178). This indicated that the* A. cepa *root tip cells can recover after an acute exposure and the toxicity can be reversible. Conversely, the chronic exposure had a much significant impact on the mitotic activities in the treated samples. All the treated samples showed depressed mitotic activity compared to the control as revealed in Table 3. Similarly, in the test for micronucleus as shown in Table 4, we recorded significant defect only in the 1*μ*g/ml and 0.5*μ*g/ml dose but not in the 0.25*μ*g/ml.

### 3.3. DHAP Induced Chromosome Aberrations

The percentage chromosome aberration induced by DHAP in root tip cells of* A. cepa *after acute exposure was indicated on Tables 5, 6, and 7.  Table 5 shows acute effect of DHAP 12hr and 24hr postexposure. A significant reduction in chromosome aberration was recorded 48hr after acute exposure as shown in Table 6. However, a robust percentage of chromosome aberrations was scored in the 48hr chronic exposure assay as indicated in Table 6. Similarly, the micronucleus test as shown in Table 7 revealed only two slides with the presence of micronucleus at 24h postexposure, but this is not significant after considering the number of other slides viewed. No micronucleus was detected 44h postexposure. However, we recorded different chromosome aberrations in this test.

**Figure 2:**
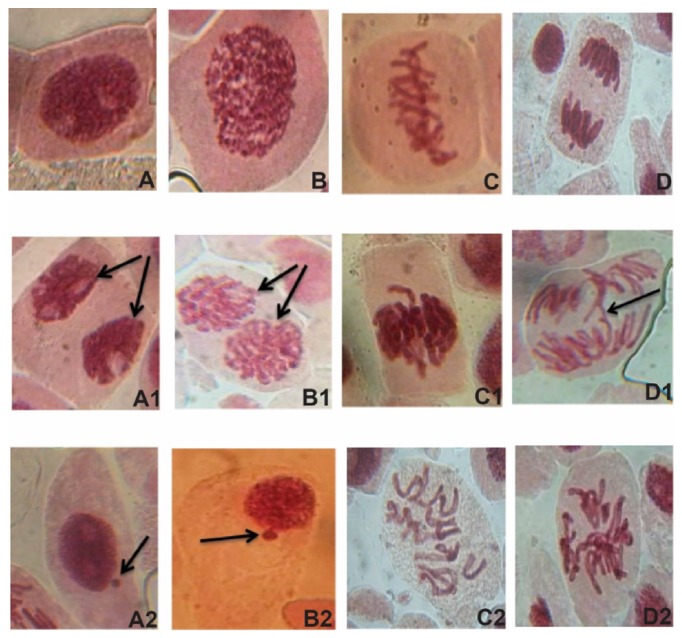
Micrographs of stages observed after exposure of A. cepa root tip meristematic cells to DHAP. A: normal interphase, A1: binuclei at interphase, A2: micronucleus at interphase, B: normal prophase, B1: binuclei at prophase, B2: micronucleus at prophase, C: normal metaphase, C1: sticky metaphase, C2: C-mitosis, D: normal anaphase, D1: anaphase bridge, and D2: multipolar.

The result revealed that most of the aberrations observed were at the metaphase and anaphase stage. Few aberrations were recorded in the interphase, prophase, and telophase. Chromosome aberrations such as sticky chromosomes, bridges, fragments, and binuclei were observed and recorded. Chromosome fragments and bridges occurred most frequently, while other aberrations were rarely found (Figure 2). The toxicological effect of DHAP was transient and there was drastic reduction in the aberrations observed after 48h. High chromosome aberrations were recorded at 12h (4.53%) and 24 (5.3%) but low at 48h (1.05%). The aberration was dose-dependent and a significant difference (P<0.05) was observed in all the treatments when compared with the mean.

### 3.4. Root Cells Recovered after Transient Exposure to DHAP

The results observed with the recovery of root growth 24hr after acute exposure suggested that the drug could have a transient effect on the mitotic activity. In order to test this, a recovery assay was set up. Root tips were exposed to DHAP momentarily for 3hrs, 6hrs, and 48hrs and recovery was scored after 12hrs. Interestingly, a recovery was observed in the group exposed for 3hrs and allowed to recover for 48hrs as shown in Table 8. This group recorded the highest mitotic activity of 4.80% at 1.0*μ*g/ml and least chromosome aberration (1.05%). Conversely, a group was exposed for 48hrs without recovery in water. This group scored the least in cells undergoing mitosis (1.44%, 1*μ*g/ml) and robust 16.9% chromosome aberrations.

### 3.5. Micronucleus Test Revealed Defects in Chromosome Behaviour after Exposure to DHAP

The percentage chromosome aberration induced by DHAP in root tip cells of* A. cepa *in the micronucleus test was indicated in Table 7. The result revealed that most of the aberrations observed were at the metaphase and anaphase stage. Few aberrations were recorded in the interphase, prophase, and telophase. Chromosomal aberrations such as bridges, fragments, binuclei, and micronucleus were observed and recorded (Figure 2). Micronuclei were observed at the 24hr but was not significant (P>0.05). There was no significant difference (P>0.05) in the values of the chromosome aberrations observed at the 0.5*μ*g/ml and 1.0*μ*g/ml concentration for 12hr and 44hr. Binuclei cells (mean= 0.2) were detected in some of the controls used for the 44hr assay.

## 4. Discussion

The results of this study have implicated DHAP as a root growth inhibitor, mitotic-depressor and clastogenic drug. The inhibition of root growth was evoked at EC_50_ of 1*μ*g/ml. This is a strong indication of its cytotoxic effect on onion root cells, which have also been found for other antimalarial drugs. They include chloroquine [23], pyrimethamine [24], mefloquine [25], artesunate [26], and artemether [27]. The root growth in* A. cepa *has been reported to occur in the cellular differentiation region which is also known as the elongation zone [28]. Biological processes such as water uptake, nitrogen mobilization, tonoplast membrane flexibility, and increase in plasma and synthesis of sugar contribute to cellular expansion [29]. These processes are mediated and promoted by metabolites and enzymes. When these biological processes are altered, it could lead to cellular toxicity and defects in homeostatic regulation [29]. The root growth inhibition observed in this study suggests that the drug contain substances that can alter biological processes that mediate cellular processes at the elongation region.

Acute exposure to DHAP had transient effect on mitosis and the chromosome aberrations. The cells were able to exhibit recovery after the elapse of the exposure period (3hrs). This supports the report of Alin* et al., *[30] on the short half-life of dihydroartemisinin (1 to 3hrs) and its high rate of being easily metabolized [31]. However, long-term exposure to DHAP inhibited mitosis and induced significant aberrations in the chromosomes. The mitotic index values in the treatment were lower than the control. This suggests the mitotic activity is suppressed by the drug.

The reduction in mitosis as observed in this study implies that DHAP contains substances that have mitotic depressive property. This may occur by inhibiting DNA synthesis and formation of microtubule. Possibly, it could be an arrest of the 24h-cycle at G1 and G2 phases or disruption of nucleoprotein synthesis and low level of ATP to supply the energy required for spindle elongation, chromosomal movement, or microtubule formation [32]. However, these hypotheses require further investigation.

Similar to the report gathered from Bakare* et al. *[33], mitotic depression in* A. cepa* root cells was also recorded in the treatment but not observed in the control. Yao* et al*. [34] reported that dihydroartemisinin can cause disruption of the cell cycle at G2/M phase of osteosarcoma, pancreas, and leukemia cells which may have exhibited a similar mechanism of action on the root cells of* A. cepa. *As the research for safer antimalarial continues, it is crucial to understand the cues that attract mosquitoes to humans and avoid being bitten [35]. This study has shown that DHAP is a strong mitotic inhibitor and could give rise to mitotic abnormalities with increase in concentration and exposure time. Their accumulation in cells may be inhibitory to cell division and growth. This calls for caution in the administration of artemisinin combination therapy for the treatment of malaria ailment. Wide spacing of dosage is therefore suggested in order to prevent the risk of genetic damage. There is need for further investigation on artemisinin-based combination therapy employing mammalian assay systems in order to ascertain their genotoxic potentials and mechanism of action on mitotic index and chromosomal behaviour.

## Figures and Tables

**Table 1:** The 96-hour root growth inhibition test on *Allium cepa* after 96 h of exposure

	Root Length (mm), Mean±SD	
Treatment group	0h	96h
Tap Water	24±0.20	60±5.22
1.5 *μ*g/ml	24±0.18	17±0.20^*∗*^
25 *μ*g/ml	24±0.13	13±0.60^*∗∗*^
75 *μ*g/ml	24±0.17	16±0.21^*∗*^
150 *μ*g/ml	24±0.15	14±0.43^*∗∗*^

Data values are mean ± standard deviation (SD) of root lengths of five determinations per group of *Allium cepa* exposed to different concentrations of DHAP. The negative control group (0hour) has been previously grown in water to reach about 24cm length. Figures marked with asterisks are significantly different from the negative control (*∗* = p<0.05; *∗∗* = p<0.01; *∗∗∗* = p<0.001). Data was analysed by two-way ANOVA, using Tukey's test.

**Table 2:** Mean values of mitotic activities observed after acute exposure of *A. cepa* root tips to DHAP.

Concentration of	12h								24h							
DHAP (*μ*g/ml)	I	P	M	A	T	TDC	TCC	MI(%)	I	P	M	A	T	TDC	TCC	MI(%)
Control	7.0	6.8	7.0	5.6	3.8	30.2	709	4.14	4.4	6.2	7.4	11.2	3.2	32.4	632	5.12
0.25	3.4	5.8	5.8	6.2	1.2	22.4	598	3.75	5.6	4.8	9.6	11.4	2.0	33.4	600	5.57
0.5	1.6	1.6	5.4	4.2	1.0	13.8	608	2.27^*∗*^	1.8	2.8	5.2	6.8	1.2	17.8	605	2.94^*∗*^
1	1.6	2.4	3.4	5.4	1.8	14.6	655	2.22^*∗*^	1.0	4.2	5.4	7.8	0.6	19.0	629	3.02^*∗*^
Mean	3.4	4.2	5.4	5.4	1.9	20.3	642	3.09	3.2	4.5	6.9	9.3	1.7	25.7	616	4.16
SD	2.52	2.53	1.50	0.84	1.28	7.84	50.8	0.99	2.16	1.41	2.06	2.35	1.12	8.39	16.3	1.38

Note: interphase (I), prophase (P), metaphase (M), anaphase (A), telophase (T), total dividing cells (TDC), total cells counted (TCC), mitotic index (MI), hour (h), and standard deviation (SD). One-way ANOVA analysed by Bonferroni multiple comparisons. MI (%) marked with asterisks are significantly different from the negative control (*∗* = p<0.05).

**Table 3:** Mean values of mitotic activities observed after long-term acute and chronic exposure of *A. cepa* root tips to DHAP.

Concentration of DHAP (*μ*g/ml)	48h (Acute)								48h (Chronic)							
	I	P	M	A	T	TDC	TCC	MI(%)	I	P	M	A	T	TDC	TCC	MI(%)
Control	7.0	6.8	7.0	5.6	3.8	30.2	709	5.26	5.2	2.2	11.2	11.4	2.2	32.2	546	5.90
0.25	1.6	8.0	14.6	15.6	2.6	42.4	646	6.60	5.2	2.4	8.0	4.4	1.2	21.2	610	3.48^*∗*^
0.5	1.2	4.2	14.0	10.4	6.4	36.2	651	5.41	2.6	1.6	5.4	6.6	1.0	17.2	626	2.74^*∗*^
1	0.8	3.2	12.0	11.0	4.4	31.4	648	4.80	1.8	1.0	4.6	1.6	0.2	9.2	638	1.44^*∗*^
Mean	2.65	5.5	11.9	10.6	4.3	35.1	663	5.31	3.7	1.8	7.3	6.0	1.2	19.9	605	3.39
SD	2.9	2.3	3.45	4.09	1.59	5.54	30.4	1.00	1.76	0.82	2.97	4.14	0.82	9.57	40.9	1.87

Note: interphase (I), prophase (P), metaphase (M), anaphase (A), telophase (T), total dividing cells (TDC), total cells counted (TCC), mitotic index (MI), hour (h), and standard deviation (SD). One-way ANOVA analysed by Bonferroni multiple comparisons. MI (%) marked with asterisks are significantly different from the negative control (*∗* = p<0.05).

**Table 4:** Mean values of mitotic activities observed in the micronucleus test after exposure of *A. cepa* root tips to DHAP.

Concentration of DHAP (*µ*g/ml)	24h								44h							
	I	P	M	A	T	TDC	TCC	MI(%)	I	P	M	A	T	TDC	TCC	MI(%)
Control	2.0	5.4	10.4	11.8	2.2	31.8	562	5.71	2.8	3.2	8.8	12.2	1.8	28.8	520	5.56
0.25	5.2	1.6	11.2	13.4	1.8	33.2	623	5.32	3.6	2.4	10.2	8.6	0.4	25.2	598	4.21
0.5	1.2	2.0	5.2	10.6	1.0	20.0	616	3.24^*∗*^	3.4	1.8	6.6	7.0	0.4	19.2	645	2.98^*∗*^
1	1.4	2.2	5.2	9.0	0.8	18.6	619	3.00^*∗*^	1.8	2.0	3.4	4.0	0.6	11.8	597	1.98^*∗∗*^
Mean	2.45	2.8	8	11.2	1.45	25.9	605	4.32	2.9	2.35	7.25	7.95	0.8	21.3	590	3.69
SD	1.86	0.99	3.24	1.86	0.66	7.66	28.8	1.39	0.81	0.62	2.96	3.41	0.67	7.44	51.7	1.55

Note: interphase (I), prophase (P), metaphase (M), anaphase (A), telophase (T), total dividing cells (TDC), total cells counted (TCC), mitotic index (MI), hour (h), and standard deviation (SD). One-way ANOVA analysed by Bonferroni multiple comparisons. MI (%) marked with asterisks are significantly different from the negative control (*∗* = p<0.05; *∗∗* = p<0.01).

**Table 5:** Mean values of chromosome aberrations observed after acute exposure of *A. cepa* root tip cells to DHAP.

Concentration of DHAP (*μ*g/ml)	12h											24h										
	S	C-M	Br	M	F	BN	MCN	AC	TCC	DC	CA (%)	S	C-M	Br	M	F	B	MCN	AC	TCC	DC	CA (%)
Control	0	0	0	0	0	0	0	0	710	41.6	0.00	0	0	0	0	0	0	0	0	632	33	0.00
0.25	0.2	0	0	0	0	0	0	0.2	598	23.4	0.85	0	0	0	0	0.2	0.2	0	0.4	601	32	1.25^*∗*^
0.5	0	0	0.2	0	0.8	0	0	1.0	607	13.0	7.69^*∗*^	0	0	0	0	0.6	0.2	0	0.8	605	17.8	4.49^*∗∗*^
1	0	0	0.6	0	0.6	0.2	0	1.4	655	14.6	9.59^*∗*^	0	0	2	0	0.6	0.4	0	3.0	629	19.2	15.6^*∗∗∗*^
Mean	0.1	0	0.3	0	0.5	0.1	0	0.9	643	23.2	4.53	0	0	0.5	0	0.35	0.2	0	1.1	617	25.5	5.3
SD	0.1	0	0.3	0	0.4	0.1	0	0.7	51.5	13.1	4.82	0	0	1	0	0.3	0.2	0	1.34	16	8.11	7.1

Note: sticky (S), C-mitosis (CM), bridge (Br), fragment (F), binuclei (BN), micronuclei (MCN), aberrant cells (AC), total cells counted (TCC), dividing cells (DC), chromosome aberration (CA), hour (h), and standard deviation (SD). One-way ANOVA analysed by Bonferroni multiple comparisons. CA (%) marked with asterisks are significantly different from the negative control (*∗*= p<0.05;*∗∗* = p<0.01;*∗∗∗*= p<0.001).

**Table 6:** Mean values of chromosome aberrations observed after long term acute and chronic exposure of *A. cepa* root tip cells to DHAP.

Concentration of DHAP (*μ*g/ml)	48h (Acute)											48h (Chronic)										
	S	C-M	Br	M	F	BN	MCN	AC	TCC	DC	CA (%)	S	C-M	Br	M	F	B	MCN	AC	TCC	DC	CA (%)
Control	0	0	0	0	0	0	0	0	656	23.8	0.00	0	0	0	0	0	0	0	0	667	25.4	0.00
0.25	0	0	0	0	0	0	0	0	646	42.4	0.00	0	0	0.6	0	1	0	0	1.6	610	21.2	7.55^*∗*^
0.5	0	0	0.6	0	0	0	0	0.6	651	36.8	1.63^*∗*^	0	0	1.4	0	0.4	0	1	2.8	626	17.2	16.3^*∗∗*^
1	0	0	0.2	0	0.6	0	0	0.8	648	31.0	2.58^*∗*^	0	0	0.4	0	3.6	0	0.2	4.2	638	9.6	43.8^*∗∗∗*^
Mean	0	0	0.2	0	0.2	0	0	0.35	650	33.5	1.05	0	0	0.6	0	1.3	0	0.3	2.15	635	18.4	16.9
SD	0	0	0.3	0	0.3	0	0	0.41	4.34	7.97	1.28	0	0	0.6	0	1.6	0	0.48	1.78	24.1	6.73	19.1

Note: sticky (S), C-mitosis (CM), bridge (Br), fragment (F), binuclei (BN), micronuclei (MCN), aberrant cells (AC), total cells counted (TCC), dividing cells (DC), chromosome aberration (CA), hour (h), and standard deviation (SD). One-way ANOVA analysed by Bonferroni multiple comparisons. CA (%) with different letters are significantly different from the negative control (*∗*= p<0.05;*∗∗* = p<0.01;*∗∗∗*= p<0.001).

**Table 7:** Mean values of chromosome aberrations observed in the micronucleus test after exposure of A. cepa root tip cells to DHAP.

Concentration of DHAP (*μ*g/ml)	24h											44h										
	S	C-M	Br	M	F	BN	MCN	AC	TC	DC	CA (%)	S	C-M	Br	M	F	B	MCN	AC	TCC	DC	CA (%)
Control	0	0	0	0	0	0	0	0	563	31.6	0.00	0	0	0	0	0	0.2	0	0.2	516	28.6	0.69
0.25	0	0	0.4	0	0.2	0	0	0.6	623	33.2	1.81^*∗*^	0	0	0.2	0	0.6	0.0	0	0.8	598	25.2	3.18^*∗*^
0.5	0	0	0.2	0	1.0	0.6	0.2	2.0	616	20.0	10.0^*∗∗*^	0	0	0.6	0	1.0	0.6	0	2.2	645	19.2	11.5^*∗∗*^
1	0	0	1.0	0	0.6	0.6	0	2.2	619	18.6	9.59^*∗∗∗*^	0	0	0	0	0.6	0.8	0	1.4	597	11.8	11.9^*∗∗∗*^
Mean	0	0	0.4	0	0.4	0.3	0.05	1.2	605	25.9	5.35	0	0	0.2	0	0.6	0.4	0	1.2	589	21.2	6.80
SD	0	0	0.43	0	0.41	0.35	0.1	1.1	28.3	7.61	5.18	0	0	0.3	0	0.4	0.36	0	0.9	53.6	7.73	5.73

Note: sticky (S), C-mitosis (CM), bridge (Br), fragment (F), binuclei (BN), micronuclei (MCN), aberrant cells (AC), total cells counted (TCC), dividing cells (DC), chromosome aberration (CA), hour (h), and standard deviation (SD). CA (%) marked with asterisks are significantly different from the negative control (*∗*= p<0.05;*∗∗* = p<0.01;*∗∗∗*= p<0.001).

**Table 8:** Normal mitotic activity and chromosome behavior can be recovered after short-term exposure to DHAP.

Duration of exposure	Recovery time in water	Mitotic index (%)				Chromosome aberrations (%)
		Control	0.25*μ*g/ml	0.5*μ*g/ml	1.0*μ*g/ml	
0hr	0hr	5.90	-	-	-	-

3hrs	12hrs	-	3.75	2.27	2.24	4.53
	24hrs	-	5.57	2.94	3.02	5.30
	48hrs	-	6.51	5.41	4.80	1.05

6hrs	24hrs	-	5.32	3.24	3.00	5.35
	44hrs	-	4.21	2.98	1.98	6.82

48hrs	0hr	-	3.48	2.74	1.44	16.9

Data was analysed with regular two-way ANOVA. Data is significant different based on duration of exposure (P<0.0001) and concentrations of DHAP (P<0.001).

## Data Availability

The data for our cell counts used to support the findings of this study are available from the corresponding author upon request.
